# Hydroxypropylation of Polyphenol-Rich Alkaline Extracts from *Pinus radiata* Bark and Their Physicochemical Properties

**DOI:** 10.3390/molecules27249002

**Published:** 2022-12-17

**Authors:** Sung Phil Mun

**Affiliations:** Department of Wood Science and Technology, Jeonbuk National University, Jeonju 54896, Republic of Korea; msp@jbnu.ac.kr

**Keywords:** *Pinus radiata* bark, alkaline extract, propylene oxide (PO), hydroxypropylation, molar substitution (MS), PEG#400

## Abstract

*Pinus radiata* bark is a rich source of polyphenols, which are mainly composed of proanthocyanidins. This study aimed to utilize *P. radiata* bark as a polyol source for bio-foam production in the future. Polyphenol-rich alkaline extracts (AEs) from *P. radiata* bark were prepared by mild alkaline treatment and then derivatized with propylene oxide (PO). Hydroxypropylated alkaline extracts (HAEs) with varying molar substitutions (MS 0.4–8.0) were characterized by FT-IR, NMR, GPC, TGA, and DSC. The hydroxyl value and solubility in commercial polyols were also determined. The molecular weights of the acetylated HAEs (Ac-HAEs) were found to be 4000 to 4900 Da. Analyses of FT-IR of HAEs and ^1^H NMR of Ac-HAEs indicated that the aromatic hydroxyl groups were hydroxypropylated and showed an increase in aliphatic hydroxyl group content. The glass transition temperature (T_g_) of AE and HAEs were 58 to 60 °C, showing little difference. The hydroxyl value increased as the hydroxypropylation proceeded. Although salts were produced upon neutralization after hydroxypropylation, HAEs still showed suitable solubility in polyether and polyester polyols; HAEs dissolved well in polyether polyol, PEG#400, and solubility reached about 50% (*w*/*w*). This indicated that neutralized HAEs could be directly applied to bio-foam production even without removing salts.

## 1. Introduction

South Korea relies on imports for about 85% of its domestic timber demand. In 2018, the total timber imports were approximately 2 million m^3^, of which over 75% constituted *Pinus radiata* wood [1]. In general, pine bark accounts for 9% to 15% of pine wood and is used as a fuel and mulch [2]. Pine barks are usually considered a low-value-added material. Therefore, a new method of utilization to increase their value is deemed necessary.

Recently, *P. radiata* bark extracts have received considerable attention as health supplements [3,4,5,6,7], cosmetics [8,9], and medicine [10,11] due to their physiological activity. In addition, pine bark extracts could be used not only as a natural brown dye, but also as a black dye through combination with various iron salts [12,13].

**Figure 1:**
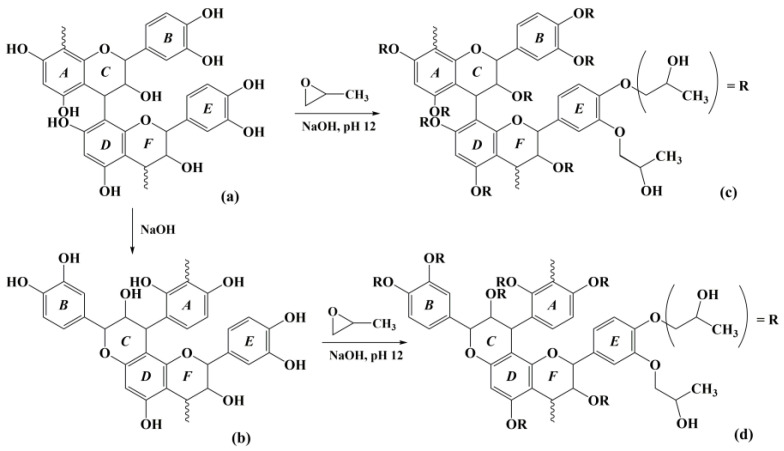
The structures of proanthocyanidin (PA) in pine bark (**a**), phlobaphene (**b**), and hydroxypropylated PA (**c**) and phlobaphene (**d**).

*P. radiata* bark is known as a rich source of polyphenols. About 50% to 60% of the bark are polyphenols, mainly composed of proanthocyanidins (PAs). PAs from *P. radiata* bark are composed of catechin-based (C15) polymers mainly linked through C4-C8 bonds and comprised of considerable amounts of hydroxyl group (Figure 1a) [4]. The inherent structural properties of these polyphenols originating from the bark render them as suitable substitutes for polyols used in the industrial production of bio-foams. Indeed, a variety of approaches have been employed to add value to wood and bark extracts, i.e., in foam manufacturing [14,15,16].

The transition from petroleum-based materials to lignocellulosic biomass-based materials, such as bark and wood wastes, would contribute greatly to the economy and the environment in countries such as Korea, where the majority of wood is imported. The development of cost-effective methods for the extraction of polyphenols from pine bark is essential.

Previously, the authors reported the optimal conditions for mass production of a polyphenol-rich fraction from *P. radiata* bark through a mild alkaline extraction using sodium hydroxide [2]. However, when the AEs prepared at the optimal condition were neutralized and then dried, the AEs were hardly soluble in commercial polyols, as well as polar organic solvents. The reason for this poor solubility was thought that polyphenols (mainly PAs), the main component of *P. radiata* bark, ionize and rearrange during alkaline extraction, and convert into phlobaphenes (Figure 1b), which are hardly soluble in polar solvents [17,18]. This low solubility may cause lowered reactivity of AEs with isocyanate in the production of polyurethane foam. Mild alkaline extraction is one of the economical methods that can easily extract these natural polyols from the pine bark in large quantities. However, the solubility of AEs in commercial polyols was still low [2]. This makes AE difficult to use as a starting material for manufacturing foams such as polyurethane. Therefore, the method of increasing the solubility of these hardly soluble phlobaphenes formed during mild alkaline extraction is not only a very important step, but also would be of great help to extend the use of pine bark polyphenols.

Recently, García et al. [19,20] have dissolved condensed tannins prepared from pine bark in alkaline solution, adjusted the pH to 12, and treated with various amounts of PO at room temperature for hydroxypropylation. They reported that the hydroxypropylation occurred easily in condensed bark tannins even under such mild conditions, and urethane foam could be manufactured using this method. Based on these studies, the author firstly investigated whether AEs from *P. radiata* bark can be converted to HAEs by PO. In addition, physicochemical characterization and thermal analysis of the HAEs were performed for future applications in bio-foam production.

## 2. Results and Discussion

### 2.1. Chemical Composition of P. radiata Bark

Previously, Ku et al. [5] studied the chemical composition of barks obtained from various *Pinus* species and reported that pine barks contained a higher percentage of polyphenols and extractives than pine wood. Table 1 shows the chemical composition of *P. radiata* bark. *P. radiata* bark used in this study also showed high contents of cold water and hot water, especially the 1% NaOH extract (68.77%). The total lignin content of *P. radiata* bark was 72.12%. This unusually high value is attributed to the polyphenols in the bark. During the determination of Klason lignin, the bark polyphenols underwent condensation reactions with strong concentrated acid (72% H_2_SO_4_) to produce phlobaphenes, which are insoluble compounds. Thus, the overestimated lignin content of the bark can be corrected by performing alkaline extraction to remove alkali-soluble polyphenols in the bark [21]. After removing the polyphenols by 1% NaOH extraction, the Klason lignin content was remarkably reduced to 13.78% compared to the initial Klason lignin (71.12%). This huge difference was due to the bark polyphenols turning to insoluble compounds, as mentioned above. Alternatively, the difference between Klason lignin (1) and Klason lignin after alkaline extraction (2) can be used as an estimation of the total polyphenol content. Based on these calculations, the total polyphenol content in the bark was 57.34%, and this result indicates that 83% of the 1% NaOH extract is composed of polyphenols.

### 2.2. Hydroxypropylation of Alkaline Extracts (AEs)

Hydroxypropylation of condensed tannins with propylene oxide (PO) under mild alkaline conditions (pH 12) has been recently performed by some researchers [19,20,22]. The etherification reaction by PO is known to be a particularly attractive reaction that dramatically affects the properties of pine bark tannins [19]. Condensed tannins are one type of polyphenol and the most abundant source of natural aromatic macromolecules after lignin [19]. Pine bark polyphenols are highly soluble in alkaline media, and most of the polyphenols are extracted with 1% NaOH, as shown in Table 1. In our previous work, when a NaOH dosage of 17–18% to *P. radiata* bark was used, an AE equivalent to 85–87% of 1% NaOH extract could be obtained, and the pH value of the extract ranged from 12 to 13 [2]. That is, under these conditions, not only could most of the polyphenols of pine bark be extracted, but an extract with a pH suitable for hydroxypropylation could be obtained. Therefore, in this study, a NaOH dosage of 17.5% to the bark was used for hydroxypropylation, and the yield of polyphenol-rich AE at this alkaline extraction condition was 59%, with a pH of 12.66.

**Figure 2:**
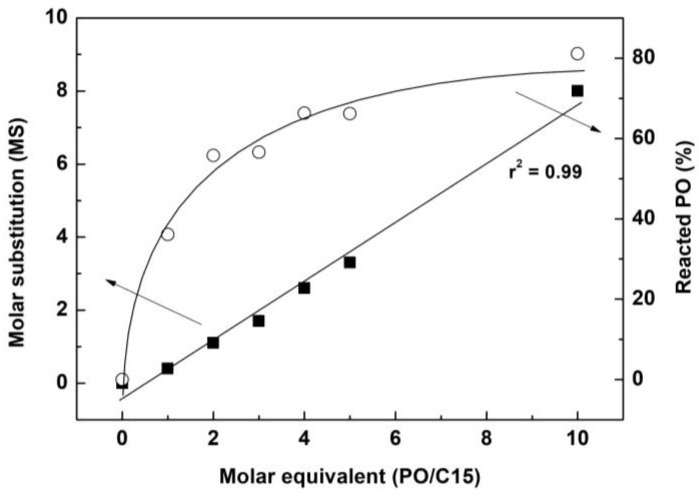
Relationship between molar substitution (MS) vs. molar equivalent (PO/C15) (filled squares) and reacted PO vs. molar equivalent (empty circles). C15: number of carbons in catechin, basic unit of proanthocyanidins in *P. radiata* bark.

Hydroxypropylation was performed by adding various amounts of PO with respect to the number of hydroxyl groups in the extract. For the hydroxypropylation of the polyphenol-rich AE, it was assumed that most of the *P. radiata* bark AE consists of proanthocyanidins (PAs), wherein the basic unit of these PAs is catechin (C15). This assumption was based on the total polyphenol content in the bark shown in Table 1. In addition, the authors’ previous studies reported that most of the hot water and neutral extracts from *P. radiata* bark were composed of PAs and that the repeating unit is procyanidin-like catechin [4,7]. Of course, it is unreasonable to assume that all AEs used in this study are composed of catechin since polyphenols in the bark undergo chemical changes during the alkaline extraction process. However, this was an inevitable assumption for the determination of the amount of PO for hydroxypropylation. The amount of PO used in this experiment was based on the total number of hydroxyl groups of catechin, which contains 5 hydroxyl groups. The hydroxypropylation was performed by adding 1 to 5 times mol PO with respect to the number of moles of hydroxyl groups in a catechin unit. In addition, the reaction in the presence of excess amounts of PO was also conducted with the addition of 10 times mol PO. Table 2 shows the result of molar substitution (MS) and reacted PO after hydroxypropylation of AE. In HAE-1, the lowest molar equivalent (PO/C15 = 1), the reacted PO was only 36.1%; however, in HAE-2 (PO/C15 = 2), the reacted PO remarkably increased to 55.8%. Thereafter, hydroxypropylation increased gradually, reaching 81.1% for HAE-10. The low hydroxypropylation value of HAE-1 was thought to be due to the low reaction frequency of the hydroxyl groups with PO. Figure 2 shows the linear relationship between MS and molar equivalent (PO/C15). However, all MS values in this study were not only lower than those of the molar equivalent value but also lower than previous reports [19,20]. The reason for these low MS values was due to the low solid content (5.91%), mostly polyphenols, in AE used in this study. This solid content was much lower than that of the solid content (20%) used by Garcia et al. [19,20]; hence the reaction frequency between PO and polyphenols was reduced. Therefore, in order to increase the reaction rate of PO, it is necessary to increase the concentration of polyphenols in AE.

Most of the HAEs prepared after hydroxypropylation were solid after drying. However, HAE-10, the one with the highest molar equivalent, was a viscous liquid.

### 2.3. FT-IR Spectroscopy

**Figure 3:**
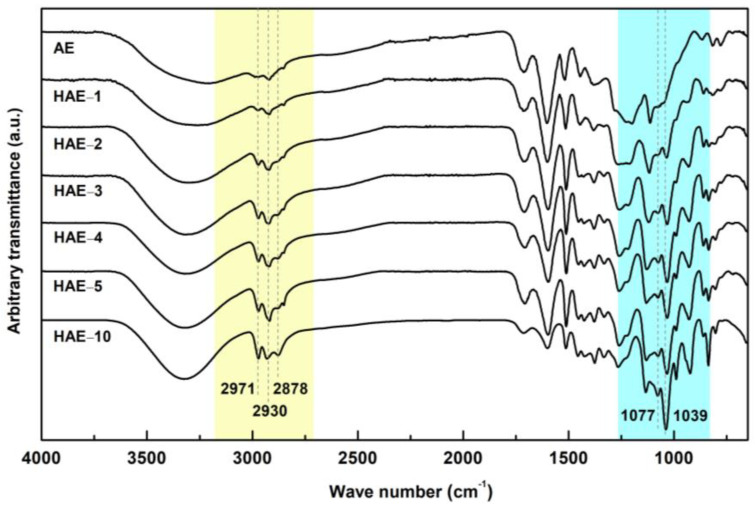
FT-IR spectra of AE and HAEs.

IR spectroscopy provides much information about the types of functional groups and their relative amounts. In this study, since an aliphatic hydroxypropyl group is newly introduced through a reaction with PO and the hydroxyl group of the bark polyphenols constituting the main component of AE, the increase in the substitution amount of the hydroxypropyl group can be qualitatively confirmed by the IR spectrum analysis. Figure 3 shows the IR spectra for AE and HAEs with various MS. The absorption bands at 3000–2800 cm^−1^ are known as the sp^3^-hybridized C-H stretching vibrations for methyl, methylene, and methine. The absorption bands at 2971, 2930, and 2878 cm^−1^ gradually increased as hydroxypropylation increased, as shown in Figure 3. In general, the C-H stretching absorption of the methyl group appears at about 2962 cm^−1^ (asym) and 2872 cm^−1^ (sym), and the methylene group appears at 2926 cm^−1^ (asym) and 2853 cm^−1^ (sym) [23]. Thus, it was predicted that the increased strength of the 2971, 2930, and 2878 cm^−1^ bands in HAEs was mainly due to the newly introduced methyl and methylene group by hydroxypropylation. The absorption band of 1300–1000 cm^−1^ is due to the vibration of C-O. 1-hexanol, a primary alcohol, has a C-O absorption at 1058 cm^−1^, and cyclohexanol and secondary alcohols have absorption bands of 1070 cm^−1^ and 1060 cm^−1^, respectively [23]. Thus, it was predicted that the increased strength of 1077 and 1039 cm^−1^ bands in HAEs was mainly due to the presence of primary and secondary aliphatic alcohols by hydroxypropylation. As a result of IR spectrum analysis, it was confirmed that the introduction of new aliphatic hydroxypropyl groups was due to the reaction of PO with polyphenols of pine bark. However, since IR analysis does not provide an accurate amount of the introduced hydroxypropyl groups, ^1^H NMR analysis was performed after acetylation to confirm this.

### 2.4. ^1^H NMR Spectroscopy

**Figure 4:**
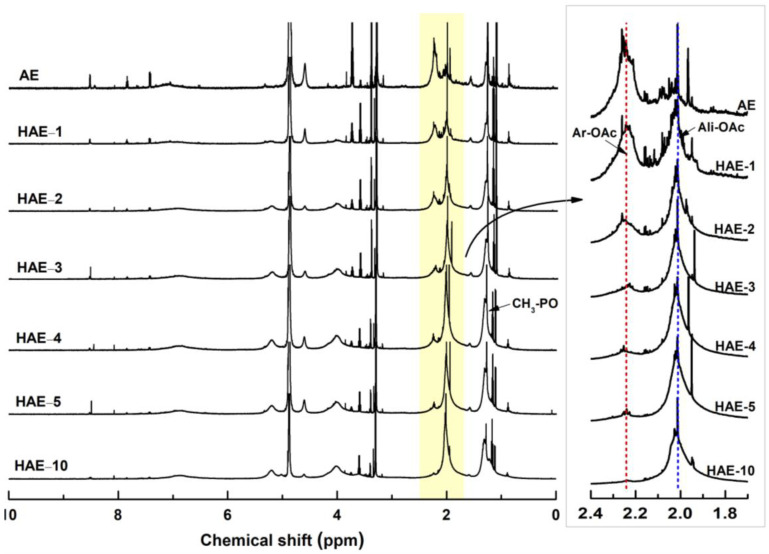
^1^H NMR spectra of acetylated AE and HAEs. Ar-OAc: aromatic acetate, Ali-OAc: aliphatic acetate.

**Figure 5:**
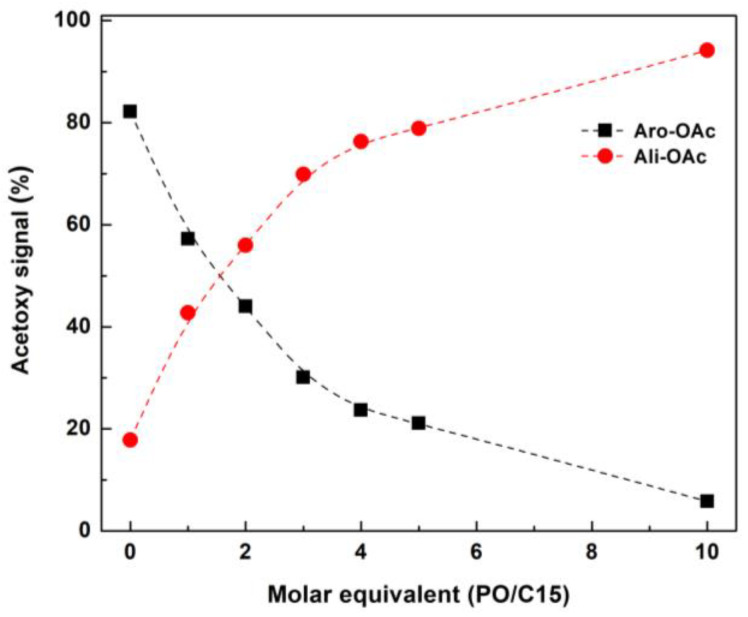
Relationship between acetoxy signal and molar equivalent of PO/C15. C15: number of carbon in catechin, basic unit of proanthocyanidins in *P. radiata* bark.

Figure 4 shows the ^1^H NMR spectra of acetylated HAEs (Ac-HAEs). Acetyl groups were introduced into aliphatic and aromatic hydroxyl groups in HAEs through acetylation. When Ac-HAEs were analyzed by ^1^H NMR, protons constituting the aliphatic and aromatic acetyl groups were clearly distinguished on the spectrum. This allows us to estimate the hydroxypropyl groups introduced to aliphatic and aromatic hydroxy groups present in AE. In Figure 4, enlarged spectra (right) of the acetoxy proton region of Ac-HAEs were included. The peaks shown at 2.1 ppm and 2.3 ppm are from acetoxy proton signals constituting aliphatic and aromatic acetyl groups, respectively. As the hydroxypropylation in AE proceeds, the intensity of the aromatic acetoxy signal in the spectrum decreases while the aliphatic acetoxy signal increases. In the case of HAE-10, AE reacted with an excess amount of PO, most of the aromatic acetoxy signal disappeared, and only the aliphatic acetoxy signal was present, indicating that most aromatic hydroxyl groups reacted with PO. The 2.0 ppm peak detected together with the aliphatic acetoxy signal on the ^1^H NMR spectrum was excluded from the calculation when designating the integration range. The peaks at 1.2–1.5 ppm are associated with the methyl group of the hydroxypropyl group introduced into AE. In order to quantify the increase or decrease in the above-mentioned hydroxyl groups, the integration value of these acetoxy signals was calculated, and the changes in the amount of these hydroxyl groups according to hydroxypropylation of AE are shown in Figure 5.

As the molar equivalent (PO/C15) was increased, the amount of aliphatic hydroxyl groups introduced increased. In the case of HAE-5, it was confirmed that about 80% of hydroxyl groups were converted to aliphatic hydroxyl groups. In the hydroxypropylation of AE shown in Table 2, the reacted PO in HAE-5 showed 66%. Assuming that most AEs are composed of proanthocyanidins, which are composed of catechin as a repeating unit, wherein a molecule of catechin has four phenolic hydroxyl groups and one aliphatic hydroxyl group. HAE-5 with a reacted PO of 66% by hydroxypropylation indicates that 3.3 out of the 5 hydroxyl groups in the catechin unit were hydroxypropylated, and MS of the hydroxypropyl group was found to be 3.3. However, as a result of ^1^H NMR of acetylated HAE-5 (Ac-HAE-5), since 80% of the aromatic hydroxyl groups were hydroxypropylated, which means that about 80% of the 4 phenolic hydroxyl groups of the catechin units constituting AE were hydroxypropylated.

### 2.5. Molecular Weight Distribution

**Figure 6:**
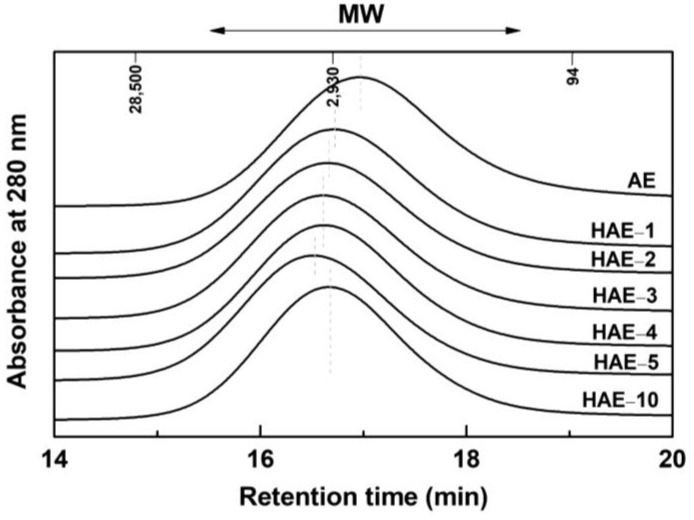
GPC chromatogram of Ac-AE and Ac-HAEs.

The AE, obtained from pine bark by weak-alkaline extraction, had poor solubility in polar solvents except with aqueous alkaline solvents. This is due to proanthocyanidins, the main component of the bark polyphenol, which has been structurally changed to insoluble phlobaphenes. For the determination of molecular weight (MW) distribution and average molecular weight, AE was dissolved in acetylation solvents. However, AE was difficult to dissolve in these solvents. In addition, the acetylated AE (Ac-AE) showed poor solubility in tetrahydrofuran (THF), the solvent used in the GPC analysis. Therefore, in GPC analysis, the THF-insoluble portion of Ac-AE was removed, and the THF-soluble portion was only subjected to the analysis. As shown in Figure 6, the overall MW distribution of Ac-AE had a higher proportion of low-molecular-weight fractions than that of Ac-HAEs. This result is attributed to the THF-insoluble portion in Ac-AE was not reflected in the molecular weight distribution. This indicates that the accurate MWs of AE are difficult to obtain due to phlobaphenes in AE.

On the other hand, HAEs showed suitable solubility in acetylation solvents. In particular, Ac-HAE-1, the one with the lowest MS (MS = 0.4), dissolved well in alcohols and acetylation solvents. Although phlobaphenes were formed during the alkaline extraction of the bark, the newly introduced aliphatic hydroxyl groups improved the solubility of HAEs.

Table 3 shows the values for $\overset{—}{M}$w, $\overset{—}{M}$n, and polydispersity of Ac-AE and Ac-HAEs. $\overset{—}{M}$n and $\overset{—}{M}$w of Ac-HAEs were slightly higher than those of Ac-AE. However, there was no remarkable difference in MW according to MS in Ac-HAEs. That is, $\overset{—}{M}$w ranged from 4000 Da to 4900 Da, and $\overset{—}{M}$n ranged from 1100 Da to 1400 Da. Polydispersity index (PDI) values were 3.0 to 3.7, also showing a small difference according to MS.

### 2.6. Thermal Analysis

**Figure 7:**
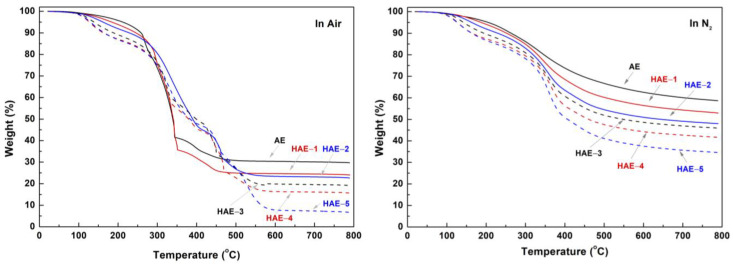
TGA of AE and HAEs in oxidative (air) and inert (N_2_) conditions.

TGA and DSC were performed to examine the thermal properties of AE and HAEs. In this study, HAEs contained a certain amount of NaCl as a neutralization product after hydroxypropylation. NaCl was also present in AE since AE was also neutralized with HCl. In order to determine the amount of inorganic components in AE and HAEs, TGA was first conducted under oxidizing conditions (air). In Figure 7, AE contained about 30% ash at temperatures above 600 °C. Most of this inorganic component was determined to be NaCl. However, the calculated amount of NaCl in AE was 25%, 5% lower than that of ash content obtained after TGA. In the case of HAEs, the ash content decreased as hydroxypropylation proceeded. For example, in HAE-5, with an MS of 3.3, the ash content was only 7–8%. This result indicates that NaCl, formed after neutralization, relatively decreased due to the increase in introduced hydroxypropyl groups.

TGA was also performed under a nitrogen atmosphere. In general, when organic substances are thermally treated under a nitrogen atmosphere, thermal decomposition and carbonization occur, leaving carbonized materials. The temperature at which carbonization of lignocellulosic biomass begins is above 400 °C, and at temperatures above 600 °C, carbonization is almost completed. The TGA curves of AE and HAEs also showed similar trends to those of lignocellulosic biomass. The residual contents of all samples at temperatures 300 °C to 500 °C were remarkably reduced due to the thermal decomposition reaction, which leads to the removal of H_2_O, CO_2_, CO, and NO_x_. At temperatures 600 °C or higher, the residual contents were almost constant, which indicates that pyrolysis was finished. The residual content decreased with increasing hydroxypropylation. This result is due to the increase in the introduced hydroxypropyl groups. That is, HAE-5 showed the lowest residual content of about 35% at 600 °C, whereas AE showed the highest residual content of about 60% due to NaCl and ash, which originated from the bark at this temperature. The corrected carbonization yield was calculated from the differences in the residue at 800 °C from oxidizing (air) and inert (N_2_) conditions. The corrected carbonization yields ranged from 28% to 30%, showing slight differences.

**Figure 8:**
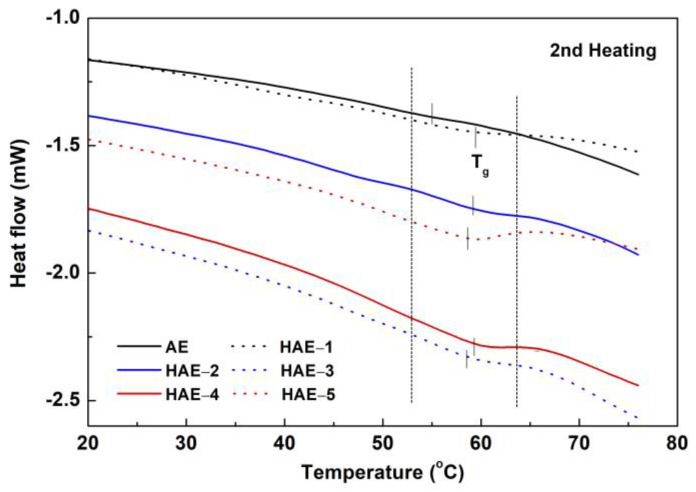
Differential scanning calorimetry thermograms of AE and HAEs at 2nd heating.

DSC analysis of AE and HAEs was also conducted. First heating, cooling, and secondary heating were performed from −80 °C to 80 °C. Although not shown here, the trends in the heating and cooling curves of the HAEs used in this experiment were similar to each other. Figure 8 shows the second heating curves of AE and HAEs. Table 4 summarizes the results of the DSC analysis of AE and HAEs. As hydroxypropylation increased, the hardening temperature increased from 29.9 °C in AE to 44.0 °C in HAE-5. During the secondary heating of HAEs, the T_g_ was in the range of 58.3–59.6 °C with no significant difference, but the T_g_ values were about 4–5 °C higher than that of AE.

### 2.7. Hydroxyl Value

**Figure 9:**
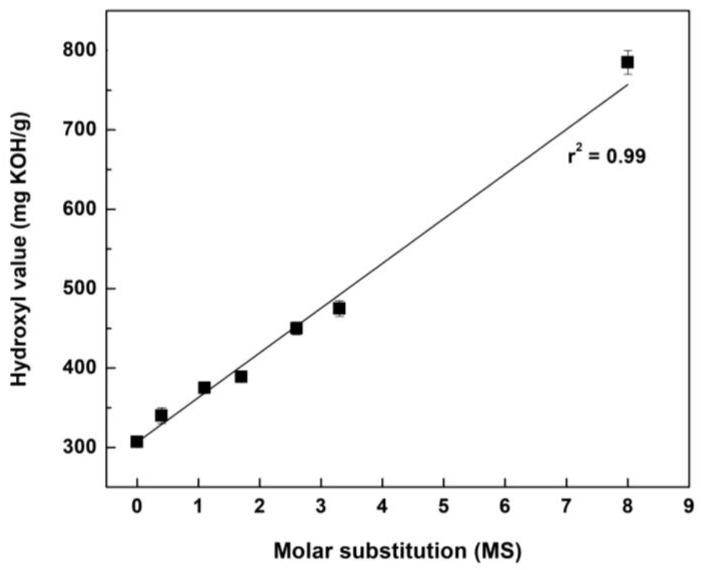
Hydroxyl value on various MS.

As mentioned in the introduction, the final goal of this work is to manufacture a polyurethane-based bio-foam from pine bark polyphenols as a natural polyol source. The number of reactive hydroxyl groups in the polyols directly affects the physical properties of the final polyurethane product. In addition, the amount of isocyanate used to prepare the polyurethane foam is determined by the hydroxyl value of the polyol. Therefore, the determination of hydroxyl value in polyols is an important step. Measurement of hydroxyl value is determined by the amount of KOH consumed through alkaline hydrolysis after acetylation of polyol. Table 5 shows the hydroxyl values of AE and HAEs. The hydroxyl value of AE was 307 mg KOH/g, and the hydroxyl value of HAE-1 to HAE-5 increased as hydroxypropylation proceeded. In the case of HAE-10, which was prepared by using an excess amount of PO, the hydroxyl value reached 785 mg KOH/g, about 2.5 times higher than that of AE. The relationship between hydroxyl value and MS had a linear correlation, as shown in Figure 9.

### 2.8. Solubility in Polyols

For AE and HAEs, solubility tests were conducted on PEG#400, a commercial polyether polyol, and SP320, a polyester polyol. AE hardly dissolved in PEG #400, as expected. However, HAE-1, the lowest hydroxypropylated sample (MS = 0.4), dissolved well in PEG #400. This increase in solubility for PEG#400 is probably due to the inhibition of condensation reaction or molecular rearrangement of bark polyphenols as AEs were hydroxypropylated. HAEs, except for HAE-10, showed suitable solubility up to 50% (*w*/*w*) in PEG#400. HAE-10, prepared with excess amounts of PO, was not solid but a highly viscous liquid. HAEs were also tested for solubility in polyester polyols (SP320) used for manufacturing polyurethane foam for panels. HAEs dissolved up to 30% in SP320, but the reaction with isocyanate was considered to be difficult to practically apply in an industrial setting due to its high viscosity. However, at 20% concentration, the reaction was sufficiently feasible. From the above results, it was concluded that flexible and rigid polyurethane foams could not only be manufactured through hydroxypropylation of AEs prepared from pine bark but can also be substituted with 50% of polyether polyol or 20% of polyester polyol.

## 3. Materials and Methods

### 3.1. Materials

*P. radiata* bark was collected from the lumberyard of Unid Co., Ltd. (Gunsan, Korea), and the scales and inner barks were removed using a cutter knife. The refined bark was crushed and then pulverized into a powder of 1 mm or less using a high-speed mill. This bark powder was placed in a zipper bag and kept in a refrigerator until further use.

Sodium hydroxide (NaOH, 93%, Duksan Pure Chemicals, Ansan, Korea) was used for the preparation of polyphenol-rich AEs from the bark, and propylene oxide (PO, 99.5%, Daejung Chemical & Metals, Shiheung, Korea) was used for the hydroxypropylation of bark AEs. Concentrated HCl (35.0%, Duksan Pure Chemicals, Ansan, Korea) was used for acidification after hydroxypropylation. Acetic anhydride (93.0%, Duksan Pure Chemicals, Ansan, Korea) and pyridine (99.5%, Kanto Chemical, Tokyo, Japan) were used for acetylation. The deuterated solvent used was CD_3_OD (Eurisotop, Saint-Aubin, France). For the determination of hydroxyl value, a standard solution of 0.5 N KOH was prepared, and a standard solution of 0.5 N HCl was purchased from Daejung Chemical & Metals (Shiheung, Korea). A polyether polyol, polyethylene glycol #400 (PEG #400, Yakuri Pure Chemicals Co., Ltd., Kyoto, Japan), and a polyester polyol, SP320G (Seho Tech, Iksan, Korea), were used for the solubility tests of hydroxypropylated alkaline extracts (HAEs).

### 3.2. Chemical Composition of P. radiata Bark

The ash, cold water, hot water, 1% NaOH, and total lignin content of *P. radiata* bark were determined according to TAPPI test methods [24,25,26,27,28]. The lignin content of the bark obtained via the Klason method is usually overestimated due to its high polyphenol content. Therefore, in this study, the bark polyphenols were first extracted with 1% NaOH and then the polyphenol-corrected lignin content was determined using the residues following 1% NaOH extraction.

### 3.3. Preparation of Alkaline Extract (AE)

A 100 g (based on oven-dried weight (o.d.)) of *P. radiata* bark powder (1 mm passed) and 500 mL of a 3.5% NaOH aqueous solution were taken into a 1-L flat bottom round flask. The flask was fitted to a condenser and placed in a PEG #400 bath preset at 110 °C. The extraction was carried out for 1 h from the start of boiling with shaking intervals every 10 min. Afterward, the bark slurry was filtered through two 17G3 glass filters with approximately equal amounts, and the residue in each glass filter was washed with 500 mL of hot distilled deionized water (DDW). The filtrates and the washings were adjusted to 1-L with DDW. The residues in the glass filter were then dried in a convection oven at 105 °C for 48 h. The yield of alkaline extract (AE) was calculated as follows:

(1) $$\mathrm{Yield}\;\mathrm{of}\;\mathrm{AE}\;\left(\%\right)=\frac{W_{o}\;\left(o.d.\right)-{\;W}_{r}\;\left(o.d.\right)}{W_{o}\;\left(o.d.\right)}\times 100$$

where W_o_ (o.d.) = oven-dried weight of pine bark powder used and W_r_ (o.d.) = oven-dried weight of residue after alkaline extraction.

### 3.4. Hydroxypropylation of AE

The combined 1-L AE, which has a pH of 12.66, was partitioned into seven beakers with 100 mL each (containing a solid content of 5.91 g (20.4 mmol, based on catechin unit)), and then a stoichiometric amount of propylene oxide (PO) was added accordingly. The amount of PO added was based on the total number of hydroxyl groups in catechin (C15, MW 290.27 g/mol; total of 5 hydroxyl groups, one aliphatic and four aromatic hydroxyl groups), which is the basic unit of proanthocyanidins (PAs), the main polyphenols present in *P. radiata* bark [5]. Thus, it was assumed that most of the polyphenols constituting the bark AE had a repeating structure of catechin units, and hydroxypropylation was performed by adding 1 to 5 times mol PO with respect to the number of moles of hydroxyl groups in a catechin unit. In addition, a reaction in the presence of excess amounts of PO was conducted with the addition of 10 times mol PO.

AEs were hydroxypropylated with various amounts of PO at ambient temperature (20–21 °C) for 24 h on a six-way magnetic stirrer (SR-306, Advantec Toyokaisha, Tokyo, Japan). After the reaction, the flask was transferred to a water bath at 60 °C for 30 min, shaken once every 10 min, and then immersed in cold running water. Afterward, the products were acidified by adding conc. HCl in a dropwise manner. The acidified samples were transferred to a 1-L oval-shaped round bottom flask. The remaining residues in the flask were dissolved with a small amount of ethanol and transferred to the flask, and then the collected samples were completely concentrated at 60 °C using a rotary evaporator. After concentration, the hydroxypropylated AEs were vacuum-dried in the presence of phosphorus pentoxide for 48 h. The hydroxypropylated alkaline extracts were abbreviated as HAEs. Reacted PO and molar substitution (MS) were calculated as follows:

(2) $${\%\;\mathrm{PO}}_{\mathrm{reacted}}=\frac{{\mathrm{HAE}}_{\mathrm{corr}}-\;\mathrm{AE}}{{\mathrm{PO}}_{i}}\times 100$$

where HAE_corr_ = corrected HAE (wt. HAE–wt. NaCl, g), HAE = wt. HAE with NaCl, g, AE = alkaline extract, g, PO_i_ = wt. propylene oxide introduced, g, and

(3) $$\mathrm{MS}=\frac{({\mathrm{HAE}}_{\mathrm{corr}}-\mathrm{AE})\times 290.27}{\mathrm{AE}\;\times 58.08}$$

where MS = molar substitution, 290.27 g/mol = MW of catechin, and 58.08 g/mol = MW of PO.

### 3.5. Acetylation of AE and HAEs

Seven samples (the neutralized AE and six HAEs: HAE-1, HAE-2, HAE-3, HAE-4, HAE-5, and HAE-10) with different MS were subjected to acetylation. A total of 100 mg of each sample and 2 mL of anhydrous pyridine were added to a 15 mL vial and then sonicated for 1–1.5 min to dissolve the sample. Then, 2 mL of acetic anhydride was added into the vial and left at ambient temperature (18–21 °C) for 48 h. The sample was shaken occasionally to aid in sample dissolution. The acetylated sample was sprayed using a tapered Pasteur pipette into a beaker containing 35 g of ice and 40 g of DDW and stirred for 1 h. The precipitates were filtered using a nylon 66 membrane filter with a diameter of 47 mm (0.45 μm, Alltech, Deerfield, IL, USA) and thoroughly washed with DDW until the odor of acetic acid was completely removed. The precipitates collected were air-dried and then vacuum-dried overnight in the presence of phosphorus pentoxide.

### 3.6. FT-IR

FT-IR analysis was performed on samples (AE, HAE-1, HAE-2, HAE-3, HAE-4, HAE-5, HAE-10) using an FT-IR spectrometer (Frontier, Perkin Elmer, Waltham, MA, USA) by using the ATR method at the Center for University-wide Research Facility, Jeonbuk National University (CURF, JBNU).

### 3.7. ^1^H NMR

Seven mg each of acetylated samples (Ac-AE, Ac-HAE-1, Ac-HAE-2, Ac-HAE-3, Ac-HAE-4, Ac-HAE-5, Ac-HAE-10) and 0.35 mL of CD_3_OD were put in a 10 mL conical beaker. The beaker was sonicated for about 1–2 min for complete dissolution and/or dispersion of the sample. Then, the filtrate, obtained by passing the sample through a filter made by packing fine glass fibers inside the Pasteur pipette, was received directly into the NMR tube. Approximately 0.4 mL of fresh CD_3_OD was poured into a conical beaker for rinsing and then transferred to the tapered Pasteur pipette for filtration to dissolve the portion remaining on the filter. The rinsed solution was transferred to the NMR tube. ^1^H NMR analysis was performed using an NMR spectrometer (600 MHz, JNM-ECA600, JEOL Ltd., Tokyo, Japan) at CURF, JBNU.

### 3.8. Molecular Weight Analysis

A 2 mg of acetylated sample and 1 mL of tetrahydrofuran (THF, 99.9%, Duksan Pure Chemicals, Ansan, Korea) were taken into a 10 mL conical beaker. The beaker was sonicated for 5 s and then filtered through a 4 mm diameter nylon syringe filter (0.45 μm, Phenomenex, Torrance, CA, USA), and the filtrate was transferred into a 2 mL vial. Gel permeation chromatography (GPC) was carried out under the conditions shown in Table 6.

### 3.9. Thermogravimetric Analysis (TGA) and Differential Scanning Calorimetry (DSC)

The non-acetylated samples (AE, HAE-1, HAE-2, HAE-3, HAE-4, HAE-5) were ground into a very fine powder using an agate mortar and then vacuum-dried to thoroughly remove moisture before thermal analysis. TGA and DSC were performed using SDT-Q600 (TA Instruments Ltd., New Castle, DE, USA) and TA Q20 (TA Instruments Ltd., New Castle, DE, USA), respectively. In TGA, the sample was heated from 24 to 800 °C at 10.00 °C/min under air and nitrogen. In the case of DSC, the sample was heated from −80 °C to 80 °C at a rate of 10.00 °C/min, followed by a cooling and second heating cycle. The glass transition temperatures (T_g_) were taken at the mid-point of the second heating cycle.

### 3.10. Hydroxyl Value

A 0.2 g each of AE and HAEs (HAE-1 to HAE-10) and 5 mL of acetylating reagents (pyridine:acetic anhydride, 4:1) were placed into a 20 mL vial. The vial was sonicated for 1 min and then placed in a convection oven preset to 95 °C for 1.5 h with shaking every 20 min. After the reaction, 1 mL of DDW was added using a micropipette, shaken, and left at ambient temperature for 10 min. The mixture in the vial was transferred to a 50 mL flask using 5 mL of acetone. The acetylated sample was titrated with a 0.5 N KOH standard solution using phenolphthalein as an indicator. During titration, the pH value was also monitored using a pH meter. From the relationship curve of the pH value with respect to the appropriate amount of KOH, the volume of the standard KOH solution consumed at pH 9 was checked, and the hydroxyl value was obtained by substitution to the formula below. A blank was tested in the same manner as above,

(4) $$\mathrm{Hydroxyl}\;\mathrm{value}=\frac{\left(\mathrm{mL}\;\mathrm{of}\;\mathrm{KOH}\;\mathrm{in}\;\mathrm{blank}\;-\;\mathrm{mL}\;\mathrm{of}\;\mathrm{consumed}\;\mathrm{KOH}\;\mathrm{in}\;\mathrm{sample}\right)\times \;f\;\times 56.11}{\mathrm{wt}.\;\mathrm{of}\;\mathrm{sample}}$$

where f = dilution factor and 56.11 g/mol = MW of KOH.

### 3.11. Solubility Test

A total of 0.5 g of sample powder was taken to a 10 mL vial and placed in a dry bath (Thermolyne DB28125, Marshall Scientific, Hampton, NH, USA) preset to 140–148 °C. Afterward, PEG #400 or SP320G was added to the vial little by little. The solubility of each polyol was visually checked while continuously adding the polyol.

## 4. Conclusions

Alkaline extracts (AEs), containing polyphenols as their main components, were prepared under mild alkaline extraction conditions, and the AEs were reacted with PO to obtain hydroxypropylated AEs (HAEs) with various molar substitution (MS). The physicochemical properties of the prepared HAEs were investigated by means of various analytical techniques. Hydroxypropylation of AE by PO was confirmed by FT-IR and ^1^H NMR spectroscopy. The molecular weight and polydispersity index of HAEs were 4000–4900 Da and 3.0–3.7, respectively, showing slight differences. The hydroxyl value of HAEs was directly proportional to the amount of PO used. In the case of HAE-10, wherein an excess amount of PO was used, the hydroxyl value was 785 mg KOH/g, about 2.5 times higher than that of AE, and was in a viscous liquid state. As a result of the solubility test of HAEs with commercially available polyester and polyether polyols, HAEs were dissolved 20% to 50% in these polyols, respectively. In conclusion, HAEs were considered a potential substitute for petrochemical-based polyols for the manufacture of polyurethane foams.

## Figures and Tables

**Table 1:** Chemical composition of *P. radiata* bark.

Ash (%)	0.77
Extracts (%)	
Cold water Hot water 1% NaOH Alcohol-benzene	21.42 31.37 68.77 12.69
Lignin	
Klason (1)	71.12
Acid soluble Total	1.00 72.12
Lignin after 1% NaOH extraction (%, based on bark)	
Klason (2)	13.78
Acid soluble Total	0.13 13.91
Total polyphenol (%) = 1–2	57.34

**Table 2:** Result of molar equivalent, molar substitution (MS), and reacted PO of HAEs.

Sample	Molar Equivalent (PO/C15)	Molar Substitution (MS)	Reacted PO (%)
HAE−1	1	0.4	36.1
HAE−2	2	1.1	55.8
HAE−3	3	1.7	56.6
HAE−4	4	2.6	66.3
HAE−5	5	3.3	66.2
HAE−10	10	8.0	81.1

**Table 3:** $\overset{—}{M}$w, $\overset{—}{M}$n, and polydispersity of Ac-AE and Ac-HAEs.

	AE	HAE−1	HAE−2	HAE−3	HAE−4	HAE−5	HAE−10
$\overset{—}{M}$w	3120	4079	4185	4376	4439	4897	4000
$\overset{—}{M}$n	792	1106	1309	1186	1353	1422	1336
$\overset{—}{M}$w/$\overset{—}{M}$n (PDI)	3.9	3.7	3.2	3.7	3.3	3.4	3.0

**Table 4:** Results of hardening temperature and glass transition temperature (T_g_) of AE and HAEs at 1st and 2nd heating.

Sample	Temperature, °C		
	T_g_, 1st Heating	Hardening Temp. after Cooling	T_g_, 2nd Heating
AE	49.42	29.92	54.10
HAE−1	54.89	38.83	59.56
HAE−2	50.68	41.72	59.57
HAE−3	50.51	42.46	58.34
HAE−4	53.52	43.66	59.01
HAE−5	55.97	43.99	58.82

**Table 5:** Hydroxyl values of AE and HAEs.

	AE	HAE−1	HAE−2	HAE−3	HAE−4	HAE−5	HAE−10
Hydroxyl value (mg KOH/g)	307 ± 2	340 ± 10	375 ± 5	389 ± 6	450 ± 8	475 ± 10	785 ± 15

**Table 6:** Analysis conditions for GPC.

GPC Configuration	LC-20AD (Pump) + SPD-M20A (Detector) + CTO-20A (Column Oven), Shimadzu, Japan
Column	PLgel 10 µm MIXED-B (300 × 7.5 mm, Agilent, Santa Clara, CA, USA)
Flow rate	0.5 mL/min
Sample injection volume	10 μL
Eluent	THF
Column oven temperature	30 °C
Detector	UV (254 nm: polystyrene standards, 280 nm: samples and phenol)

## Data Availability

Not applicable.
